# Dietary patterns of US Chinese Older Adults

**DOI:** 10.1093/geroni/igab046.3587

**Published:** 2021-12-17

**Authors:** Alexander Dong, Lisa Lanza, Qun Le, Stephanie Bergren, Dana Dychtwald

**Affiliations:** 1 Rutgers University, New Brunswick, New Jersey, United States; 2 Rutgers University, Rutgers Institute for Health, New Jersey, United States

## Abstract

A westernized diet, characterized by intake of foods high in fat and sugar, has been associated with several chronic conditions. However, the dietary pattern of US Chinese older adults is not well understood. The primary objective of this study was to determine the relationship between years of US residence and other demographic factors and the intake of foods high in fats and sugar. As part of the Population Study of Chinese Elderly, participants were given a 48-item food frequency questionnaire, which were further placed into primary food groups. Each group was then categorized into whether they consumed the food group at least once a week. The total sample was 59% female with an average age of 75, with 49% consuming fatty foods and 85% consuming sweets in the past week. Using logistic regression (N=3053), each additional year of US residence (range of 0-93 years) was associated with a higher dietary intake of fats (OR: 1.01 (95%CI:1.01-1.02)) and sweets (OR: 1.01 (95%CI:1.00-1.02)). Additionally, higher education was associated with lower consumption of fats (OR: 0.98 (95%CI:0.96, 0.99)) and higher income was associated with higher consumption of fats (OR: 1.11 (95%CI: 1.04, 1.18)). For sweets, women compared to men were 54% less likely to consume sweets in the last week (OR: 0.46 (95%CI:0.36, 0.59)), and higher education was associated a greater likelihood of consuming sweets (OR: 1.07 (95%CI: 1.05, 1.10)). Study findings suggest that immigration related factors and demographic factors may influence consumption of a westernized diet high in fats and sugars.

